# Comparison of laboratory testing methods for the diagnosis of tuberculous pleurisy in China

**DOI:** 10.1038/s41598-017-04872-6

**Published:** 2017-07-03

**Authors:** Qing Zhang, Caicun Zhou

**Affiliations:** 1 Soochow University, Suzhou, 215006 China; 2 Tuberculosis Clinic and Research Center, Shanghai Key Lab of Tuberculosis, Shanghai Pulmonary Hospital, Tongji University School of Medicine, Shanghai, 200433 China; 3 Department of oncology, Shanghai Pulmonary Hospital, Tongji University School of Medicine, Shanghai, 200433 China

## Abstract

To evaluate the diagnostic utilities of different methods for *Mycobacterium tuberculosis* (*M. tuberculosis*) detection in pleural fluid that represent potentially cost-effective measures for resource-limited settings in China. We compared diagnostic characteristics of the AmpSure simultaneous amplification and testing method, the BACTEC MGIT 960 system, and acid-fast bacilli staining of specimen smears for detection of *M. tuberculosis* in pleural fluids of 438 patients with suspected tuberculous pleurisy. Chest radiographs, computed tomography scans and the results of sputum and pleural biopsy testing were used for confirmations of tuberculosis diagnoses. The sensitivity of the AmpSure test (21.2%) was significantly higher than that of smear analysis (3.1%; p < 0.001), but was similar to that of the BACTEC culture method (17.8%; p > 0.05). The specificity of the AmpSure, BACTEC, and smear tests were 100%, 96.4%, and 100%, respectively. The positive and negative predictive values of the AmpSure, BACTEC, and smear tests were 100%/23.1%; 78.6%/19.8%; and 100%/22.4%, respectively. The sensitivity of ADA, IFNγ and histopathological analyses of pleural biopsies were all 100%. The sensitivities of all three methods were suboptimal for the detection of *M. tuberculosis* in pleural fluid. Future studies of a composite diagnostic index consisting of a combination of these tests are warranted.

## Introduction

Although the incidence of tuberculosis (TB) has decreased during the previous decade, it remains a major cause of morbidity and mortality worldwide, with the greatest disease burden occurring in Asia and Africa^1^. Tuberculous pleurisy (TP) is a common presentation of extrapulmonary *Mycobacterium tuberculosis* infection, which occurs secondary to pulmonary TB^2^. In TP patients, the rupture of subpleural caseous foci causes tuberculous pleural effusion (TPE), which most often occurs 6–12 weeks following primary *M. tuberculosis* infection, resulting in variable numbers of tubercle bacilli invading the pleural space^3, 4^. Currently available laboratory tests for *M. tuberculosis* detection are suboptimal for diagnosing pleural extrapulmonary TB^5^. The ability to rapidly detect *M. tuberculosis* in pleural fluid (PF) could provide a valuable diagnostic tool for identifying TP cases.

Bacteriological detection of *M. tuberculosis* in pleural biopsies can fail to identify up to 45% of patients with TPE^4^, and methods for culturing *M. tuberculosis* take 2–5 weeks to complete^4, 6, 7^, which can delay effective medical interventions. Approximately 33% of TP patients have a negative tuberculin skin test^4^, and as many as75%–80% of patients with TPE test negative for acid-fast bacilli (AFB) in PF smear tests^4, 8^. The detection of elevated biomarkers of *M. tuberculosis* infection in PF samples^9–11^ and the presence of parenchymal lesions associated with TP^12^ have also been investigated for TP diagnosis, but these methods require bacteriological confirmation. The Centers for Disease Control and Prevention (USA) first recommended nucleic acid amplification for TB diagnosis in 2000^13^, and the identification of *M. tuberculosis* based on RNA detection and quantification has gained favor in recent years due to lower false-positive rates^14^.

The AmpSure diagnostic test for *M. tuberculosis* (Shanghai Rendu Biotechnology, Shanghai China) is based on a previously described simultaneous amplification and testing method for *M. tuberculosis* detection^15^ that uses isothermal RNA amplification and real-time fluorescence detection. The AmpSure assay has demonstrated high levels of sensitivity and specificity for the diagnosis of pulmonary TB based on the analysis of sputum and bronchial lavage fluid^16, 17^. However, the efficacy of the AmpSure assay for detecting tubercle bacilli in PF has not been reported. Our current study aimed to evaluate the diagnostic accuracy of the AmpSure assay for identifying patients with TPE, and to optimize the sensitivity of the assay for detecting *M. tuberculosis* in PF.

## Methods

### Patients

Our study was approved by the Ethics Committee of Shanghai Pulmonary Hospital, and was performed in accordance with the Declaration of Helsinki with regard to ethical principles for research involving human subjects. Written, informed consent was obtained from all of the patients prior to participation in our study.

We prospectively screened patients who had been admitted to our hospital for suspected TP between January 2014 and April 2015. Data regarding age; sex; history of TB contacts; history of TB symptoms; current TB symptoms; history of medical treatment for TB; comorbidities; and concurrent medical therapies were obtained from each patient using a standardized questionnaire. The exclusion criteria for enrollment were as follows: <17 years of age; seropositive for HIV; differential diagnosis of transudative pleural effusion; and a history of previous medical treatment for TB. Enrolled patients for whom a clear diagnosis could not be made after examination and treatment were excluded from our analysis.

Patients were assigned to the TB or non-TB group based on a diagnosis of TB, which required chest radiography or computed tomography (CT) features consistent with fluid patterns in exudative pleural effusion^18^ and at least one of the following criteria: *M. tuberculosis* detected in PF or sputum; histological findings of caseating granulomas based on examination of pleural biopsy specimens; and the absence of other diseases that could have caused *M. tuberculosis* invasion of the PF. Patients for whom these diagnostic criteria were not satisfied were assigned to the non-TB group.

### Examinations

Each patient underwent physical examination, chest radiography or CT, and guided thoracentesis using ultrasound or CT. Pleural biopsy was performed as needed. For all PF samples, an adenosine deaminase (ADA) analysis and the blood T-SPOT.TB interferon-gamma release assay were performed, and *M. tuberculosis* detection was performed using the AmpSure assay, AFB smear, and bacteriological analysis. Sputum specimens were subjected to AFB smear and bacteriological analysis only. The diagnostic testing and histopathological analysis were performed at the TB reference laboratory in Shanghai Pulmonary Hospital using routine quality control procedures. Bacteriological analysis was performed using the BACTEC MGIT 960 system (BD Life Sciences, Franklin Lakes, NJ, USA), according to the guidelines of the World Health Organization^19^. The AmpSure assay was performed according to the manufacturer’s protocol, as previously described^16^. The ADA was analyzed using a colorimetric assay (Diazyme Laboratories, Poway, CA, USA) with a cut-off value of 40 IU/mL, and the blood T-SPOT.TB interferon-gamma (IFNγ) release assay was performed as previously described^20^, with a cut-off value for IFNγ-positive lymphocytes of 50%. Pleural biopsy specimens were examined by a qualified pathologist.

### Statistical analyses

The statistical analysis was conducted using the SPSS, version 18.0 software (IBM, Armonk, NY, USA). Continuous variables are reported as the mean ± standard deviation and range, and categorical variables are reported as the number and percentage of observations. Differences in the continuous and categorical variables were evaluated using a two-tailed Fisher exact test or a Pearson chi-squared analysis where appropriate.

The sensitivity, specificity, positive predictive value (PPV), negative predictive value (NPV), and diagnostic accuracy of the AmpSure assay for detecting *M. tuberculosis* in PF were calculated. Sensitivity was defined as the number of patients with a positive test result divided the number of patients in the TB group. Specificity was defined as the percentage of non-TB patients with a negative result. The PPV was defined as the number of TB patients with a positive result divided by the total number of positive results (true positives + false positives). The NPV was defined as number of non-TB patients with a negative result divided by the total number of negative results (true negatives + false negatives). Accuracy rate was defined as the proportion of patients for whom test results were consistent with diagnosis. Concordance between the results of the AmpSure and bacteriological analyses was assessed using the Cohen kappa test, with κ > 0.75 indicating excellent agreement, a κ-value of 0.4−0.75 indicating moderate agreement, and κ < 0.4 indicating poor agreement. The level of statistical significance was set at p < 0.05.

## Results

We prospectively enrolled 442 patients. Four patients for whom a clear diagnosis could not be determined were excluded from our analysis. The demographic characteristics of patients and study group assignment are shown in Table 1. The remaining 438 patients included 269 men and 169 women with a mean age of 47.5 ± 18.4 years, among whom 354 patients (219 men and 135 women) were confirmed TB cases and 84 patients (50 men and 34 women) had pleural effusion due to an etiology other than TB. There was no statistically significant difference in sex between the TB and non-TB groups (χ^2^ = 0.157, p = 0.692). The TB group was significantly younger (43.4 ± 18.7 years; range: 17–82 years) than the non-TB group (57.2 ± 14.6 years; range: 23−82 years; p < 0.001). Diagnoses for patients in the non-TB group included lung cancer (n = 10), invasive pulmonary fungal infection (n = 1), bacterial pneumonia (n = 65), sarcoidosis (n = 1), pulmonary embolism (n = 2), systemic lupus erythematosus (n = 1), and non-tubercle *Mycobacterium* infection (n = 4).

**Table 1:** Patient characteristics and study group assignment.

Variable	TB Group (N = 354)	Non-TB Group (N = 84)
Men (n [%])	219 (61.9)	50 (59.5)
Women (n [%])	135 (38.1)	34 (40.5)
Age (y)	43.4 ± 18.7	57.2 ± 14.6
Mt-positive sputum or pleural fluid culture (n [%])	288 (81.4)	0 (0)
Caseating granuloma in pleural biopsy (n [%])	66 (18.6)	0 (0)

TB, tuberculosis; Mt, *Mycobacterium tuberculosis*.

The results of the various diagnostics tests are summarized in Table 2.

**Table 2:** Summary of results of the diagnostic tests.

Method	Diagnostic rate (n/N)					
	Sputum*		PF		Histopathology	
	TB group	Non-TB group	TB group	Non-TB group	TB group	Non TB group
AmpSure positive	208/278	0/76	75/354	0/84		
AFB smear positive	87/278	2/76	11/354	0/84		
Mt-culture positive	245/278	0/76	63/354	0/84		
Histopathology-confirmed					66/66	12 negative/12
IFNγ release of PF (>50%)			354/354	66/84		
ADA of PF (>40 IU/L)			354/354	20/84		
AmpSure positive/AFB and Mt-culture negative			35/354	0/84		
AFB positive/AmpSure and Mt- culture negative			0/354	2/84		
Mt-culture positive/AmpSure and AFB negative			22/354	0/84		

*Sputum from 278/354 TB cases and from 76/84 non-TB cases was examined. AFB, acid-fast bacilli; PF, pleural fluid; Mt, *Mycobacterium tuberculosis*; IFNγ, interferon gamma; ADA, adenine deaminase.

In the TB group, 278 cases were also complicated by pulmonary TB based on sputum analysis. In the analysis of PF, the diagnostic rates for the AFB smear (3.1%), AmpSure assay (21.2%), and culture analyses (17.8%) were lower than that of ADA analysis (100%) and IFNγ release (100%). The diagnostic rates for the AFB smear, AmpSure assay, and culture analyses of PF were also lower than those of the AFB smear (31.3%) and culture analyses (88.1%) of sputum as well as the histopathological analysis of pleural biopsy (100%).

The comparison of the diagnostic characteristics of the various methods of *M. tuberculosis* detection is shown in Table 3. The sensitivity of the AmpSure assay for the analysis of PF samples (21.2%) was significantly higher than that of the AFB smear analysis (3.1%; χ^2^ = 54.2, p < 0.001), but it was not significantly different than the sensitivity of the BACTEC MGIT 960 testing of PF samples (17.8%; χ^2^ = 1.291, p = 0.255). Both the AmpSure assay and the BACTEC MGIT 960 system demonstrated higher specificities and PPVs for PF than those of the AFB smear analysis, and the NPVs of all of the tests were <24%. Concordance between the results of the AmpSure assay and the BACTEC MGIT 960 test for sputum for the entire cohort (N = 438) and that of the AmpSure assay and culture method for PF was moderate (κ = 0.571, p < 0.001). The results suggested that the diagnostic characteristics are suboptimal for the detection of *M. tuberculosis* in PF, and that the characteristics of the BACTEC and smear analyses likely differed between the sputum and PF samples.

**Table 3:** Comparison of AmpSure, AFB smear, and culture results for pleural fluid samples.

Method	Sensitivity	Specificiy	PPV	NPV	Accuracy
AmpSure	21.2%	100%	100%	3.1%	36.3%
AFB smear	3.1%^a^	96.4%	78.6%	19.8%	21.0%
Mt culture	17.8%^b^	100%	100%	22.4%	33.6%

AFB, acid-fast bacilli; PF, pleural fluid; Mt, *Mycobacterium tuberculosis*;

^a^p < 0.001 compared with AmpSure, ^b^p > 0.05 compared with AmpSure.

## Discussion

In this study, we evaluated three laboratory methods for the diagnosis of TPE in Chinese patients, and found that all were suboptimal for the detection of *M. tuberculosis* in PF. Similar previous investigations of methods for detecting *M. tuberculosis* in the PF have also reported suboptimal sensitivities for identifying patients with TPE^3, 4, 21, 22^. However, the AmpSure diagnostic test has demonstrated high levels of sensitivity and specificity for the detection of *M. tuberculosis* in sputum samples^16^. Therefore, in our comparison of the diagnostic utility of the AmpSure test for detecting *M. tuberculosis* in PF to that of the BACTEC MGIT 960 and AFB smear analyses, we found that the sensitivities of these methods are suboptimal for the detection of tubercle bacilli in PF samples, whereas each of them demonstrates high specificity.

The higher sensitivity of the AmpSure assay for detecting *M. tuberculosis* in sputum samples, relative to that of AFB smear analysis, suggests that it might also perform better for detecting tubercle bacilli in PF. However, studies have shown that the sensitivities of various methods of direct detection of tubercle bacilli can vary between respiratory and nonrespiratory samples, despite the levels of potential inhibitors of nucleic acid detection, such as RNAases, being present at similar levels^23, 24^. Moreover, the results of our direct comparison of AmpSure detection of the 16 S ribosomal RNA of *M. tuberculosis*, the selective propagation of *M. tuberculosis* in liquid culture medium by the BACTEC MGIT method, and the AFB smear analysis suggest that the factors which contribute to the reduced sensitivities of these methods for PF testing are nonspecific in nature.

In addition, previous studies of other molecular methods of *M. tuberculosis* detection in PF samples have reported higher sensitivities than that which we determined for the AmpSure test in our current study, with sensitivities of 25–43.6% and 93% for the Xpert MTB/RIF test^25, 26^ (Cepheid, Sunnyvale, CA, USA) and a specific antibody-based detection method^27^, respectively. In addition, one previous study of a PCR-based analysis reported a sensitivity of 93.1%^28^. However, the specific antibody-based test has not been replicated, and the PCR-based analysis and Xpert MTB/RIF evaluations included a total of only 177 PF samples, whereas our analysis included samples from 438 suspected TP cases. One possible shortcoming of our analysis was the composite index used to confirm TB diagnosis. Clinical examinations and the interpretation of chest radiographs and CT scans are subject to variation due to the expertise and experience of clinicians. However, no “gold standard” exists for the direct detection of tubercle bacilli in PF against which our results could be compared, and the TB diagnostic index we used represented an approach applicable to resource-limited settings in China.

Smear microscopy for the detection of AFB remains the most widely used diagnostic test for *M. tuberculosis* detection in resource-limited settings around the world. The cost of the Xpert MTB/RIF are prohibitive for most patients in China. The cost of the AmpSure test is, however, a reimbursable expense under China’s national insurance program^16^. A study in China found that including the BACTEC MGIT test in routine diagnostic testing for TB patients could be a cost-effective long-term measure^29^. A cost comparison showed that prices for AmpSure, BACTEC and smear test were 14 USD, 22 USD and 2 USD, respectively. Given the high specificities observed for the AmpSure test (100%), BACTEC MGIT test (100%), and AFB smear analysis (96.4%) in our current study, a composite index consisting of these tests might be the most effective strategy for the diagnosis of TP at centralized diagnostic facilities with real-time fluorescence detection instruments in China. The diagnostic rate of the combination of the above three different methods reached 79.1%. Such a composite index would represent a balance between the cost-effectiveness and diagnostic accuracy of these methods in an area where TB burden is high.
